# Indicators of Data Quality at the Cancer Registry Zurich and Zug in Switzerland

**DOI:** 10.1155/2018/7656197

**Published:** 2018-06-13

**Authors:** Miriam Wanner, Katarina L. Matthes, Dimitri Korol, Silvia Dehler, Sabine Rohrmann

**Affiliations:** ^1^Cancer Registry Zurich and Zug, Institute of Pathology and Molecular Pathology, University Hospital Zurich, Zurich, Switzerland; ^2^Epidemiology, Biostatistics and Prevention Institute, University of Zurich, Zurich, Switzerland

## Abstract

Data quality is an important issue in cancer registration. This paper provides a comprehensive overview of the four main data quality indicators (comparability, validity, timeliness, and completeness) for the Cancer Registry Zurich and Zug (Switzerland). We extracted all malignant cancer cases (excluding non-melanoma skin cancer) diagnosed between 1980 and 2014 in the canton of Zurich. Methods included the proportion of morphologically verified cases (MV%), the proportion of DCN and DCO cases (2009–2014), cases with primary site uncertain (PSU%), the stability of incidence rates over time, age-specific incidence rates for childhood cancer, and mortality:incidence (MI) ratios. The DCO rate decreased from 6.4% in 1997 to 0.8% in 2014 and was <5% since 2000. MV% was 95.5% in 2014. PSU% was <3% over the whole period. The incidence rate of all tumours increased over time with site-specific fluctuations. The overall M:I ratio decreased from 0.58 in 1980 to 0.37 in 2014. Overall, data quality of the Cancer Registry Zurich and Zug was acceptable according to the methods presented in this review. Most indicators improved over time with low DCO rates, high MV%, low PSU%, relatively low M:I ratios and age-specific incidence of childhood cancer within reference ranges.

## 1. Introduction

The Cancer Registry Zurich and Zug in Switzerland was established in 1980 and covers roughly 20% of the Swiss population (1.56 Mio of 8.19 Mio in 2014). The reporting of cancer data is compulsory in the canton of Zurich since the introduction of the cantonal law on cancer registration in 2017. Before that, several legal bases and approvals have ensured a high level of data reporting from pathology institutes, hospitals, and general practitioners. The purpose of population-based cancer registration is monitoring, epidemiological research, and health policy making. Cancer statistics depend on the quality of data assessed in cancer registries. Therefore, a good data quality is essential.

Cancer registries are encouraged to assess the quality of their data [1–3]. Several methods to report data quality have been proposed including qualitative and quantitative methods. These address the following indicators: comparability, validity, timeliness, and completeness. Comparability is the extent to which coding and classification procedures as well as definitions of recording and reporting specific items adhere to agreed international guidelines [1]. Validity (accuracy) is defined as the proportion of cases in a dataset with a given characteristic (e.g., site and age) that truly have the attribute [1]. Timeliness relates to the rapidity at which a registry can collect, process, and report sufficiently reliable and complete cancer data [1]. However, there is a trade-off between timely data and the extent to which it is complete and accurate [1]. Finally, completeness is the extent to which all of the incident cancers occurring in the population are included in the registry database [2]. Completeness is a prerequisite to present incidence rates and survival proportions [2].

Several cancer registries have reported on the quality of their data [4–15]. In Switzerland, a recent analysis has assessed completeness in ten regional cancer registries using the mortality to incidence rate ratio with relative survival (MI-Surv method) and the flow method [16]. In the Swiss study, incidence data from 2006 until 2010 were presented.

The present study aims to provide a comprehensive overview of the four main data quality indicators for the cancer registry of Zurich and Zug for the period of 1980 until 2014 including different cancer types and using a variety of methods.

## 2. Methods

### 2.1. Data Sources

Data of all cancer cases diagnosed between 1980 and 2014 in the canton of Zurich were extracted in June 2017. The focus is on the canton of Zurich (1.44 mio inhabitants in 2014) because data assessment in the canton of Zug (0.12 mio inhabitants in 2014) only started in 2011. The cancer registry Zurich and Zug receives notifications from pathology and haematology laboratories, hospitals, and physicians as well as death certificates from the Swiss Federal Statistical Office. Data include personal information and tumour characteristics. Vital status follow-up was not conducted annually, because we did not have access to vital status information from the citizen service departments. The death statistics (and death certificates) that we receive from the Swiss Federal Statistical Office once a year are anonymous and linkage with the cancer registry database is not straightforward. Since incidence year 2009, a one-year and a five-year follow-up have been carried out.

Only malignant cancer cases (C00-C99, excluding nonmelanoma skin cancer (C44)) were included. For some indicators/methods, data are presented for the whole period 1980–2014 (e.g., incidence data); for others, data are only presented for the periods 1997–2014 (death-certificate-only (DCO) cases) or 2009–2014 (death-certificate notification (DCN) cases). Distinguishing between DCN and DCO cases is only possible since 2009 due to a change in the database software. Furthermore, DCO cases were not systematically marked before 1997. For the whole period 1980–2014, 197,493 incident tumours were available. For the analyses including only the periods 1997–2014 and 2009–2014, the respective numbers were 115,947 and 43,719 tumours. For specific parameters, only the most common cancer localisations are presented (Tables 1, 2, and 4).

The Swiss Federal Statistical Office provided population and mortality data (1981–2014). The coding of the mortality data is based on the International Statistical Classification of Diseases and Related Health Problems (ICD) 10^th^ revision and conducted according to the rules defined by the WHO since 1995. Permanent resident population data, which include Swiss citizens with main place of residence in Switzerland and foreign citizens with a residence permit for at least 12 months, were used at midyear.

### 2.2. Comparability, Validity, Timeliness, and Completeness

Regarding comparability, a general description of adherence to international guidelines, standards for classification and coding of neoplasms, definition of incidence date, and rules for multiple primaries is given. Validity is represented by the proportion of morphologically verified cases (MV%, 1997–2014), the proportion of DCO (1997–2014) and DCN (2009–2014) cases, and cases with primary site uncertain (=C80 according to ICD 10, PSU%, 1980–2014). Furthermore, the procedures regarding internal consistency checks are presented. A general description of timeliness is provided in addition to the comparison of incident cases published in annual reports for specific years, indicating the proportion of cases that were registered “too late.” Regarding completeness, the following semiquantitative methods were used: the stability of incidence rates over time was investigated for specific tumour groups. Incidence rates were age-standardised using the 1976 European Standard Population [18]. Furthermore, the mortality:incidence (MI) ratios and the age-specific incidence rates per 100,000 for childhood cancer were assessed. Age-specific incidence rates for childhood cancer were calculated over the whole period 1981–2014 for the age strata 0–4, 5–9, and 10–14 years including all types of cancer. As suggested by Parkin & Bray (2009) [2], we used the reference intervals based on deciles for childhood cancer published in CI5 Volume VIII [17].

**Figure 1:**
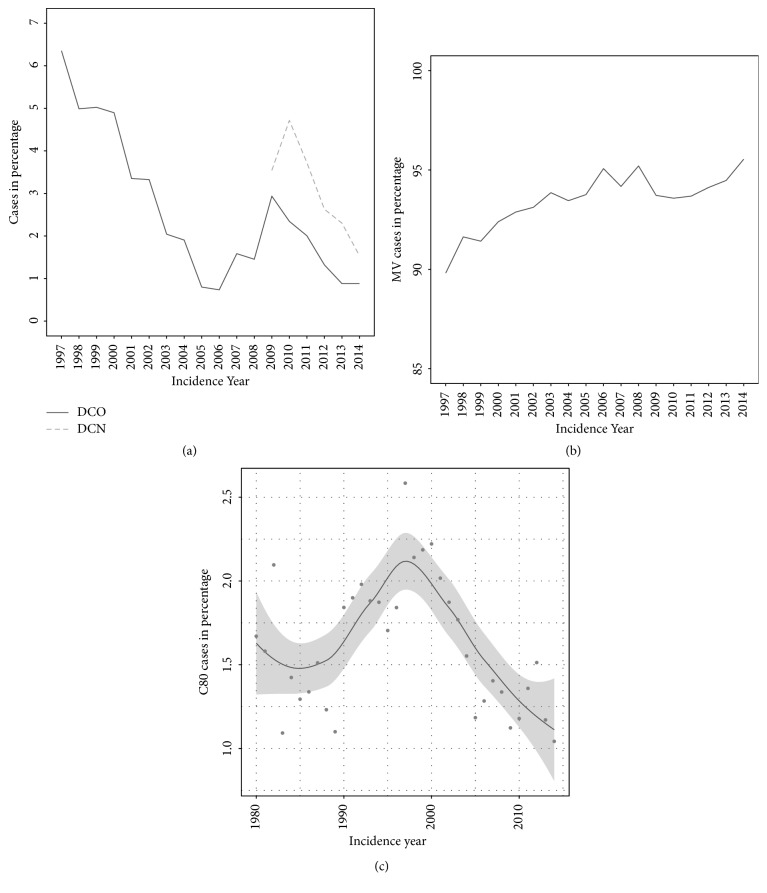
(a) Proportion of death certificate only (DCO, 1997–2014) and death certificate notification (DCN, 2009–2014) cases, (b) proportion of morphologically verified cases (MV%, 1997–2014), (c) proportion of cases with primary site unknown (PSU%, 1980–2014), Cancer Registry Zurich and Zug, Switzerland. Figure 1(c) displays a smoothed curve; the shaded area presents the 95% confidence intervals.

**Figure 2:**
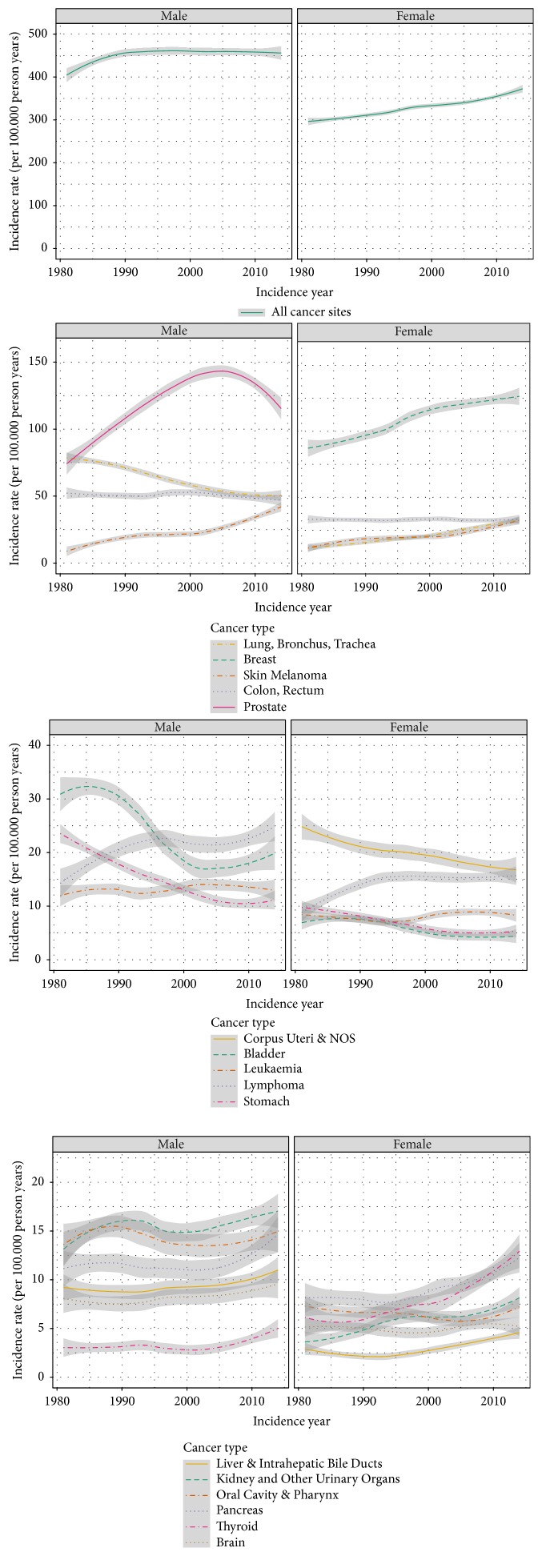
Annual trends in age-standardized incidence rates overall and for selected tumours, 1981–2014, Cancer Registry Zurich and Zug, Switzerland. The curves are smoothed; the shaded areas present the 95% confidence intervals.

Due to limitations in updated vital status information, methods including survival were not applied. All statistical analyses were performed using R Version 3.4.0. The curves in Figures 1(c) and 2 were smoothed using LOESS regression (Local Polynomial regression fitting) and the shaded areas present the 95% confidence intervals.

## 3. Results

### 3.1. Comparability

The Cancer Registry Zurich and Zug records the first occurrence of all malignant tumours (excluding nonmelanoma skin cancer) and some early forms (in situ) of selected topographies. All cancer cases are recorded and coded according to international standards. The first registration of tumours occurred according to ICD-9 for the period 1980–2002 and according to ICD-10 since 2003.

The histological classification (morphology) and topography of the tumours for the diagnosis years 1980–2002 was coded according to the first edition of the International Classification of Diseases for Oncology (ICD-O) in accordance with the codes of ICD-9. Since the diagnosis year 2003, the coding is based on the third edition of ICD-O in accordance with the codes of ICD-10. Topography codes for the period 1980–2002 were converted into ICD-10 codes for reporting purposes only. The tumour stages are coded according to the TNM system of the International Union against Cancer (UICC): TNM 3 (1980–1994), TNM 4 (1995–1997), TNM 5 (1998–2002), TNM 6 (2003–2009), and TNM 7 (since 2010).

Incidence dates are defined according to the recommendations of the European Network of Cancer Registries (ENCR). The date of histological confirmation or the date of the first pathology report confirming a cancer has the highest priority. If the clinical confirmation of the diagnosis was more than three months before the histological confirmation, the clinical date is considered the date of diagnosis.

The most valid basis of diagnosis is selected according to the recommendations of the International Agency for Research on Cancer (IARC) and the International Association of Cancer Registries (IACR) [19]. The recording of multiple primary tumours follows the recommendations of ENCR [20]. The topography codes considered as single sites and systemic and multicentric cancers were counted only once. If a new tumour (e.g., diagnosed simultaneously in the same site) has a different morphological code (e.g., the first four digits denote a different cell type), it is considered as a new cancer case.

### 3.2. Validity

The proportion of DCO cases has decreased from 6.4% in 1997 to 0.8% in 2014 with a peak of about 3% in 2009 (Figure 1(a), Table 1). DCN cases decreased from 3.6% in 2009 to 1.5% in 2014. In 1997, the DCO rate was highest for pancreatic cancer (16.9%) and carcinoma of the liver and intrahepatic bile ducts (12.2%) and lowest for skin melanoma (1.0%) and thyroid cancer (1.5%, Table 1). In 2014, the DCO rate was highest for leukaemia (2.0%) and pancreatic cancer (1.7%) and lowest for skin melanoma, tumours of the oral cavity and pharynx, and thyroid and brain cancer (0.0%). For carcinoma of the liver and intrahepatic bile ducts, the DCO rate decreased to 1.4% in 2014. The DCN rate in 2014 was highest for carcinoma of the liver and intrahepatic bile ducts (5.5%) and stomach cancer (4.8%).

MV% has increased from 89.7% in 1997 to 95.5% in 2014 (Figure 1(b), Table 2). In 1997, the proportion was lowest for pancreatic cancer (62.2%) and highest for skin melanoma (99.0%, Table 2). In 2014, the proportion was lowest for carcinoma of the liver and intrahepatic bile ducts (68.3%) and highest for skin melanoma, tumours of the oral cavity and pharynx, and thyroid cancer (100.0%). For pancreatic cancer, the proportion increased to 81.3% in 2014.

PSU% was overall low with 1.6% in 1980, 1.1% in 2014, and a peak of 2.6% in 1997 (Figure 1(c)).

After one incidence year has been completed, IARC checks as well as ENCR checks (since incidence year 2014) are performed. Any errors are checked and corrected, if applicable.

### 3.3. Timeliness

Currently, the Cancer Registry Zurich and Zug completes the incident cases with a two-year delay. That is, at the end of 2017, incident cases of 2015 are completely registered and coded. The Cancer Registry Zurich and Zug publishes annual reports since 2009. This requires “freezing” the database at a certain point in time (usually in December). For example, in December 2017, the data of incidence year 2015 are exported; these are published in spring 2018. The advantage is that most information on these cancer cases is available by the time of coding the cases. However, at the time of publishing the data, they are already somehow “out-dated” (three-year delay).

Based on the annual reports 2014–2016, Supplementary Material Table 1 presents the number of incident cases for specific localisation for the incidence years 2012 and 2013. For most localisations, the difference in cases registered within two years after diagnosis and within three years after diagnosis was less than 5% but tended to be somewhat higher for leukaemia and liver cancer. About 2.5% of all cases were registered one year later than intended.

### 3.4. Completeness

Figure 2 shows the stability of incidence rates between 1981 and 2014. Overall, the incidence of all tumours combined increased both for men and for women. For men, the most pronounced increase was between 1981 and 1990, while for women a linear increase was observed over the whole period. The annual trends for some cancer sites fluctuate, but there does not seem to be any pattern. Increasing incidence trends were observed for breast cancer and lung cancer in women and for skin melanoma in both sexes, while lung cancer in men showed a decreasing trend. Prostate cancer increased up to 2005 and decreased thereafter. The incidence of stomach and bladder cancer decreased while lymphomas increased slightly in both sexes.

Table 3 presents the age-specific incidence rates per 100,000 for childhood cancer for 1981–2014 (all sites). All values are within the reference values (upper and lower deciles for childhood cancer incidence rates published in volume VIII of CI5) [17].

The M:I ratio is displayed in Table 4. Overall, the M:I ratio decreased from 0.58 in 1980 to 0.37 in 2014. Cancers with poor survival rates (e.g., pancreas, lung, stomach, liver) had M:I ratios close to one, whereas skin melanoma had low M:I ratios of about 0.1 or 0.2 over the whole observation period.

## 4. Discussion

The present study gives an overview of the four main indicators of data quality in cancer registration (comparability, validity, timeliness, and completeness) for the Cancer Registry Zurich and Zug in Switzerland that registers data since 1980. In general, the data quality in the Cancer Registry Zurich and Zug is acceptable according to the methods presented in this study.

### 4.1. Comparability

The Cancer Zurich and Zug generally follows international standards of coding, definition of incidence date, and rules regarding multiple primaries.

### 4.2. Validity

The DCO rate decreased from over 6% to less than 1% in 2014. Internationally, DCO rates of <5% are regarded as satisfactory. The DCO rate for the Cancer Registry Zurich and Zug decreased below 5% around the year 2000 and below 1% in 2005. The increase to 3% in 2009 was triggered by limited data access to two pathology laboratories at that time (of which one delivered the reports at a later stage). However, in general, DCO rates for the Cancer Registry Zurich and Zug were in an acceptable range and have been below 5% since the year 2000.

A general increase in MV% was observed for most cancer sites in the Cancer Registry Zurich and Zug between 1997 and 2014. This indicates that a higher proportion of cancer cases was based on histology reports, as MV% generally reflects the diagnostic process.

PSU% was overall low (<3%) with a peak in 1997. The increase between 1980 and 1997 may be due to increased awareness of PSU and cancer diagnostics. The subsequent decrease may likely be due to improved diagnostic techniques that allowed for finding the primary site in a higher percentage of new cancer diagnoses (Binder et al., manuscript in preparation).

### 4.3. Timeliness

There is no formal definition of timeliness in a cancer registration context [1]. However, some standards have been set. The Centers for Disease Control and Prevention/National Programme of Cancer Registries request that, within 24 months of the close of the diagnosis year, 95% of expected unduplicated cases are available to be counted as incident cases [1]. Similarly, the North American Association of Central Cancer Registries defines this time span to be 23 months [1]. In the Cancer Registry Zurich and Zug, the difference in the number of cases reported for the incidence years 2012 and 2013 in the annual reports 2014, 2015, and 2016 is mostly smaller than 5%, indicating that 95% of cases were registered within 24 months. The proportion is specifically low for melanoma (about 1%). For leukaemia, the proportion is up to 10%, indicating that these cancers were more frequently missed within two years and we get notifications for these later on. This is in line with other research indicating that (lymphoid) leukaemia was systematically underregistered [16]. One reason could be that chronic types of leukaemia are often diagnosed in outpatient settings where the notification procedures for cancer registries are not yet well established.

A national law on cancer registration will presumably come into force in 2020. This law aims to accelerate the process of cancer registration all over Switzerland, such that, at the end of one year, the incident cases of the previous year should be completed.

### 4.4. Completeness

Overall, cancer incidence rates of men and women in the Canton of Zurich increased. Similar trends were reported for Norway [5], Iceland [6], and Finland [7]. The age-specific childhood cancer incidence rates were within the limits of the reference values, although close to the upper limit especially for boys aged 0–4 years. Other (mostly Nordic) countries have reported high rates of childhood cancer [7], which can be attributed to true variations in underlying risks [21].

The most common cancers were prostate cancer in men and breast cancer in women. While breast cancer is still increasing, prostate cancer increased up to 2005 and decreased thereafter, which probably reflects the introduction of PSA testing. An increase in the number of breast cancer cases was also observed in other countries such as Bulgaria [4], Iceland [6], Norway [5], and Finland [7]. The lung cancer trends reflect changing smoking patterns in the population, with a decreasing proportion of smokers in men and an increasing proportion in women in the last decades [22]. While the incidence of colon and rectum cancer was relatively stable over time, the incidence of skin melanoma more than doubled in the observation period. Increases have been observed in other European countries such as Italy [23], Finland [24], and Lithuania [25]. Furthermore, compared to other European countries, the incidence rate of skin melanoma is high in Zurich and Switzerland in general [26]. Comparably high rates were observed in Nordic countries such as Denmark, Norway, Netherlands, and Sweden, whereas Central, Eastern, and Southern European countries had mostly much lower rates [26]. Reasons for the high rates in Switzerland are assumed to be an increased sun exposure (due to travel behaviour favourably to sunny destinations, more frequent outdoor activities, and increased use of sunbeds) and increased dermatological consultations leading to greater awareness [27].

The site-specific M:I ratios are comparable to other European countries with values closer to one for pancreas, liver, brain, and lung cancer [5, 6]. M:I ratios >1 (e.g., for pancreatic cancer) are probably due to incorrect coding on the death certificate or can occur because the incident and the mortality cases in one calendar year are not necessarily referring to the same patients.

### 4.5. Strengths and Limitations

Strengths of the study are the presentation of a variety of methods that address data quality focusing on the four main indicators of data quality. Furthermore, due to cancer registration dating back to 1980 in the Canton of Zurich, data quality indicators were presented over time, demonstrating that the indicators improved over time and were overall in an acceptable range. A limitation is that only semiquantitative but no quantitative methods to reflect completeness as suggested by Parkin & Bray were applied [2]. Moreover, due to limited access to vital status data, no methods that are based on survival were used, such as the Flow method [3]. However, with the new cantonal law, access to vital status data will be improved. The national law that will come into force in about 2020 foresees an annual matching of the cancer registry data with mortality data of the Central Compensation Office based on the unique personal insurance number, which will considerably improve survival data in the Cancer Registry Zurich and Zug.

## 5. Conclusions

The Cancer Registry Zurich and Zug has a long experience of cancer registration starting in 1980. Overall, the access to data is relatively good and is likely to improve with the new cantonal law on cancer registration that came into force in early 2017. The adherence to international standards is good. According to the methods presented in this review, the data quality of the Cancer Registry Zurich and Zug was acceptable. Most indicators improved over time with low DCO rates, high MV%, low PSU%, and relatively low M:I ratios. In addition, age-specific incidence rates of childhood cancer were within the reference limits. A drawback is the limited access to vital status information, which poses a problem on survival analyses. However, the new cantonal law and the national law that will come into force in 2020 will certainly improve this issue. Good data quality is a prerequisite for using cancer registry data for monitoring, research, and health policy making.

## Figures and Tables

**Table 1:** Percentage of DCO (1997–2014) and DCN (2009–2014) cases, Cancer Registry Zurich and Zug, Switzerland.

	**ICD-10**	**1997**	**1998**	**1999**	**2000**	**2001**	**2002**	**2003**	**2004**	**2005**	**2006**	**2007**	**2008**	**2009**		**2010**		**2011**		**2012**		**2013**		**2014**	
		**DCO**												**DCN**	**DCO**	**DCN**	**DCO**	**DCN**	**DCO**	**DCN**	**DCO**	**DCN**	**DCO**	**DCN**	**DCO**
**Oral cavity & pharynx**	C00-C14	1.6	3.6	0.7	0.7	1.4	0.0	0.0	0.6	0.0	0.0	1.7	1.3	1.2	1.2	2.3	1.1	1.0	0.5	0.4	0.4	1.4	0.5	0.0	0.0
**Stomach**	C16	7.3	4.1	8.9	5.2	1.4	0.7	4.6	2.3	1.6	0.7	1.7	2.4	4.6	3.1	7.7	2.8	5.6	3.5	1.4	0.0	3.8	1.6	4.8	1.6
**Colon, rectum**	C18-C20	7.6	5.8	6.5	5.9	4.5	4.0	2.5	0.9	0.2	1.0	1.6	0.9	2.8	2.4	3.2	1.5	5.1	2.7	2.2	1.0	1.6	0.5	1.8	1.0
**Liver & intrahepatic bile ducts**	C22	12.2	13.6	19.5	12.3	7.4	5.2	2.1	2.9	1.1	1.1	3.6	3.2	12.9	11.2	9.7	2.6	9.6	0.0	9.8	3.5	8.6	0.0	5.5	1.4
**Pancreas**	C25	16.9	18.2	17.1	15.6	12.6	11.0	10.8	3.4	3.3	1.1	4.0	5.2	10.6	9.7	17.1	9.2	13.3	8.0	7.4	2.5	7.0	3.3	4.6	1.7
**Lung, bronchus, trachea**	C33-C34	7.5	5.4	5.5	6.7	3.3	3.9	3.1	3.9	0.6	0.8	2.0	2.0	4.3	3.9	9.5	4.0	6.9	4.3	4.1	2.2	4.1	1.2	1.9	1.1
**Skin melanoma**	C43	1.0	0.4	0.3	0.0	0.0	0.0	0.3	0.3	0.3	0.0	0.4	0.0	0.2	0.0	0.6	0.6	0.3	0.2	0.8	0.3	0.3	0.3	0.0	0.0
**Breast**	C50	5.1	2.7	2.3	3.5	3.4	3.5	1.3	1.1	1.0	0.6	0.5	1.1	1.9	1.5	1.4	1.0	0.7	0.6	1.2	0.7	0.4	0.3	0.2	0.2
**Corpus uteri & NOS**	C54-C55	3.3	3.9	3.6	1.2	2.2	1.5	0.7	0.6	0.6	0.0	0.6	0.0	2.5	1.3	2.5	1.9	2.5	0.5	0.7	0.7	1.9	1.2	1.9	1.2
**Prostate**	C61	4.6	4.3	4.3	4.8	3.8	3.8	2.0	1.6	0.7	0.6	1.3	2.0	3.3	2.7	2.5	1.4	2.4	1.5	1.3	0.6	1.9	1.2	0.9	0.8
**Kidney and other urinary organs**	C64-C66, C68	6.6	3.0	5.4	6.1	1.5	2.5	1.3	0.0	1.4	0.0	1.8	1.3	5.6	5.0	6.9	4.0	3.8	2.4	3.6	2.4	1.8	1.8	1.0	0.5
**Bladder**	C67	5.4	3.2	3.7	6.2	2.1	2.9	2.5	3.4	1.1	0.5	2.6	1.1	3.8	3.8	6.3	3.9	1.8	0.9	2.5	1.2	1.9	0.5	1.3	0.9
**Brain**	C70-C72	8.5	8.4	11.1	8.5	4.1	6.3	3.5	0.0	0.0	1.1	3.4	2.9	3.5	1.7	11.6	2.5	6.9	2.3	2.7	0.9	3.2	0.0	0.0	0.0
**Thyroid**	C73	1.5	0.0	1.3	0.0	0.0	2.6	1.4	0.0	0.0	0.0	0.0	0.9	0.0	0.0	2.7	1.8	0.0	0.0	0.0	0.0	0.0	0.0	0.0	0.0
**Lymphoma**	C81-C85, C96	3.4	1.6	3.5	3.0	1.8	1.1	0.4	1.2	0.0	0.6	1.0	0.3	2.3	2.0	2.5	1.1	1.4	0.9	1.8	1.5	0.8	0.6	1.3	1.1
**Leukaemia**	C91-C95	10.9	11.1	5.2	5.2	5.0	4.3	4.0	1.9	0.6	3.3	1.7	4.7	9.4	7.4	9.0	5.1	3.6	2.1	7.1	2.7	4.3	2.2	3.5	2.0
**All cancer sites**	C00-C99^1^	6.4	5.0	5.0	5.0	3.4	3.4	2.1	1.9	0.8	0.8	1.6	1.5	3.6	3.0	4.6	2.3	3.6	2.0	2.5	1.3	2.3	0.9	1.5	0.8

^1^Excluding C44; DCN, death-certificate notification; DCO, death-certificate-only; ICD-10, International Statistical Classification of Diseases and Related Health Problems 10^th^ revision.

**Table 2:** Percentage of morphologically verified (MV%) cases, 1997–2014, Cancer Registry Zurich and Zug, Switzerland.

	**ICD-10**	**1997**	**1998**	**1999**	**2000**	**2001**	**2002**	**2003**	**2004**	**2005**	**2006**	**2007**	**2008**	**2009**	**2010**	**2011**	**2012**	**2013**	**2014**
**Oral cavity & pharynx**	C00-C14	97.7	96.4	97.2	99.3	98.6	100.0	97.3	98.8	99.5	100.0	98.3	98.0	98.8	98.3	99.0	99.6	97.1	100.0
**Stomach**	C16	92.0	94.6	86.7	93.3	95.2	98.7	94.7	96.9	97.7	97.0	96.6	95.2	95.4	95.8	94.4	99.3	96.2	97.6
**Colon, rectum**	C18-C20	90.8	92.8	92.5	93.1	94.7	95.4	96.0	97.0	97.4	97.1	96.5	98.1	96.4	97.8	95.2	96.3	96.9	96.7
**Liver & intrahepatic bile ducts**	C22	70.3	79.0	59.7	75.3	73.4	63.9	71.9	72.5	59.1	69.2	64.5	63.2	54.3	56.1	57.0	58.0	60.9	68.3
**Pancreas**	C25	62.2	64.2	62.5	68.2	61.0	71.8	72.8	73.0	73.8	77.5	76.9	78.0	70.8	76.0	71.9	80.2	78.9	81.3
**Lung, bronchus, trachea**	C33-C34	88.7	89.8	87.8	89.2	88.0	91.1	90.3	89.4	93.0	94.0	91.3	92.1	91.1	88.1	89.3	89.8	91.5	92.6
**Skin melanoma**	C43	99.0	99.6	99.3	100.0	100.0	99.7	99.7	99.0	99.1	99.7	99.6	99.8	100.0	99.4	99.8	99.5	99.7	100.0
**Breast**	C50	94.1	96.3	96.3	95.7	95.8	95.6	96.9	97.9	97.5	98.9	99.3	98.0	97.9	98.6	98.9	98.5	99.2	99.5
**Corpus uteri & NOS**	C54-C55	95.4	95.5	94.7	97.0	97.2	98.5	97.8	98.8	98.2	100.0	97.7	99.3	96.9	97.5	97.5	96.7	97.5	98.1
**Prostate**	C61	90.7	91.0	92.0	91.8	92.0	91.8	92.8	92.1	91.6	93.8	93.7	93.8	93.7	94.4	93.6	93.5	93.2	95.3
**Kidney and other urinary organs**	C64-C66, C68	84.2	92.4	89.8	87.9	94.0	91.3	87.3	90.4	90.5	85.9	87.9	91.3	88.8	87.4	89.1	88.2	92.3	89.9
**Bladder**	C67	91.6	95.5	94.2	92.1	97.2	95.4	94.4	95.5	96.2	95.8	94.9	96.2	94.8	93.7	96.0	96.3	95.3	97.8
**Brain**	C70-C72	72.0	79.0	76.5	85.1	85.6	77.9	87.1	75.0	82.4	87.4	81.2	91.2	90.5	83.5	85.5	89.3	87.1	91.6
**Thyroid**	C73	98.5	100.0	98.7	100.0	98.6	97.4	98.6	100.0	100.0	100.0	100.0	99.1	100.0	97.3	100.0	99.3	100.0	100.0
**Lymphoma**	C81-C85, C96	96.6	98.4	96.1	97.0	97.9	98.1	98.5	98.4	99.3	99.1	99.0	98.7	97.4	98.2	98.9	96.5	97.8	98.4
**Leukaemia**	C91-C95	89.1	88.9	94.8	94.8	95.0	95.7	96.0	98.1	99.4	96.7	98.3	95.3	92.6	94.9	97.9	97.3	97.8	98.0
**All cancer sites**	C00-C99^1^	89.7	91.5	91.4	92.3	92.8	93.0	93.7	93.3	93.6	95.0	94.1	95.1	93.6	93.5	93.7	94.1	94.4	95.5

^1^Excluding C44. DCN, death-certificate notification; DCO, death-certificate-only; ICD-10, International Statistical Classification of Diseases and Related Health Problems 10^th^ revision.

**Table 3:** Age-specific incidence rates per 100,000 for childhood cancer (all sites) by gender, 1981–2014, Cancer Registry Zurich and Zug, Switzerland.

**Age**		**Boys**			**Girls**	
	Lowest decile^1^		Highest decile^1^	Lowest decile^1^		Highest decile^1^
0-4	<12.3	24.3	>24.7	<9.7	17.0	>21.4
5-9	<8.5	13.1	>15.6	<6.9	10.0	>12.0
10-14	<8.5	13.0	>15.0	<6.8	10.5	>13.6

^1)^The lowest and highest deciles for childhood cancer incidence rates are published in CI5 Vol. VIII [17].

**Table 4:** Mortality:incidence (M:I) ratios for selected types of cancer, 1980–2014, Cancer Registry Zurich and Zug, Switzerland.

	**ICD-10**	**1980**	**1981**	**1982**	**1983**	**1984**	**1985**	**1986**	**1987**	**1988**	**1989**	**1990**	**1991**	**1992**	**1993**	**1994**	**1995**	**1996**	**1997**	**1998**	**1999**	**2000**	**2001**	**2002**	**2003**	**2004**	**2005**	**2006**	**2007**	**2008**	**2009**	**2010**	**2011**	**2012**	**2013**	**2014**
**Oral cavity & pharynx**	C00-C14	0.4	0.6	0.5	0.6	0.4	0.6	0.4	0.5	0.5	0.5	0.4	0.4	0.4	0.3	0.4	0.4	0.3	0.3	0.4	0.3	0.4	0.3	0.3	0.2	0.2	0.4	0.3	0.3	0.3	0.2	0.3	0.3	0.3	0.3	0.3
**Stomach**	C16	0.9	0.8	0.9	0.9	0.9	0.9	0.9	0.7	0.9	0.7	0.8	0.7	0.9	0.6	0.8	0.7	0.6	0.7	0.6	0.7	0.6	0.6	0.6	0.5	0.6	0.7	0.6	0.7	0.6	0.6	0.6	0.6	0.6	0.4	0.7
**Colon, rectum**	C18-C20	0.4	0.4	0.4	0.4	0.4	0.4	0.4	0.4	0.5	0.4	0.4	0.4	0.4	0.3	0.4	0.5	0.5	0.4	0.4	0.4	0.5	0.4	0.4	0.4	0.4	0.4	0.3	0.4	0.4	0.3	0.4	0.4	0.4	0.3	0.4
**Liver & intrahepatic bile ducts**	C22	0.6	0.6	0.9	0.7	0.9	0.9	0.9	0.9	0.6	1.0	0.7	1.1	0.8	1.0	0.9	0.9	0.7	0.8	0.8	0.9	0.8	0.9	0.8	0.9	0.7	1.0	0.9	0.8	0.9	0.8	0.6	1.0	0.8	0.9	0.8
**Pancreas**	C25	1.0	1.1	1.1	0.9	1.1	1.0	1.1	1.0	1.1	1.0	1.0	0.9	1.0	1.2	1.0	0.8	1.0	0.9	0.8	0.9	0.9	1.0	1.0	0.9	0.8	0.8	0.9	0.9	0.9	0.8	0.9	0.9	0.8	0.8	0.8
**Lung, bronchus, trachea**	C33-C34	0.9	0.8	0.9	0.9	0.9	0.9	0.9	0.9	0.8	0.8	0.9	0.9	0.9	0.9	0.8	0.8	0.8	0.8	0.8	0.8	0.8	0.8	0.8	0.7	0.7	0.8	0.7	0.7	0.7	0.7	0.8	0.6	0.6	0.7	0.7
**Skin melanoma**	C43	0.1	0.1	0.1	0.1	0.1	0.1	0.2	0.1	0.1	0.2	0.1	0.1	0.1	0.0	0.1	0.2	0.2	0.2	0.2	0.1	0.2	0.1	0.1	0.1	0.2	0.1	0.1	0.1	0.1	0.1	0.1	0.1	0.1	0.1	0.1
**Breast**	C50	0.5	0.5	0.4	0.5	0.4	0.4	0.5	0.5	0.4	0.4	0.4	0.5	0.4	0.4	0.4	0.3	0.3	0.3	0.3	0.2	0.2	0.2	0.2	0.3	0.3	0.3	0.3	0.2	0.2	0.2	0.2	0.2	0.2	0.2	0.2
**Corpus uteri & NOS**	C54-C55	0.6	0.7	0.8	0.7	0.7	0.6	0.6	0.6	0.6	0.5	0.6	0.6	0.7	0.8	0.7	0.7	0.7	0.8	0.7	0.6	0.5	0.5	0.7	0.7	0.5	0.5	0.8	0.6	0.7	0.6	0.6	0.5	0.6	0.5	0.6
**Prostate**	C61	0.5	0.5	0.5	0.5	0.5	0.4	0.4	0.4	0.4	0.4	0.5	0.3	0.3	0.3	0.3	0.3	0.3	0.3	0.3	0.3	0.2	0.3	0.2	0.2	0.2	0.2	0.2	0.2	0.2	0.2	0.2	0.2	0.2	0.2	0.2
**Kidney and other urinary organs**	C64-C66, C68	0.7	0.5	0.5	0.6	0.5	0.6	0.5	0.6	0.6	0.5	0.6	0.5	0.5	0.4	0.5	0.5	0.4	0.4	0.4	0.4	0.4	0.4	0.3	0.3	0.5	0.4	0.3	0.3	0.3	0.3	0.3	0.3	0.3	0.3	0.3
**Bladder**	C67	0.3	0.5	0.3	0.4	0.4	0.5	0.3	0.4	0.4	0.3	0.4	0.4	0.5	0.4	0.4	0.4	0.4	0.6	0.6	0.5	0.5	0.5	0.4	0.6	0.4	0.4	0.3	0.3	0.5	0.4	0.5	0.3	0.3	0.5	0.4
**Brain**	C70-C72	0.7	0.8	0.8	1.0	0.9	0.8	0.6	1.0	0.7	0.8	0.7	1.0	0.9	0.8	1.2	0.6	1.0	0.8	0.8	0.8	0.8	0.6	0.7	0.8	1.0	0.7	0.7	0.7	0.7	0.7	0.7	0.7	0.8	0.8	0.8
**Thyroid**	C73	0.4	0.2	0.4	0.4	0.2	0.3	0.3	0.4	0.3	0.2	0.5	0.2	0.2	0.2	0.1	0.2	0.2	0.2	0.3	0.1	0.1	0.1	0.1	0.1	0.2	0.1	0.1	0.1	0.1	0.1	0.1	0.1	0.1	0.1	0.1
**Lymphoma**	C81-C85, C96	0.4	0.6	0.7	0.7	0.5	0.5	0.5	0.6	0.5	0.5	0.5	0.4	0.4	0.4	0.5	0.5	0.4	0.4	0.3	0.4	0.3	0.4	0.4	0.3	0.4	0.3	0.3	0.2	0.3	0.3	0.3	0.2	0.3	0.3	0.2
**Leukaemia**	C91-C95	0.7	0.8	0.9	0.6	0.8	0.6	0.8	0.6	0.8	0.7	0.6	0.7	0.7	0.7	0.8	0.6	0.6	0.6	0.6	0.6	0.5	0.5	0.4	0.4	0.5	0.5	0.5	0.5	0.5	0.4	0.5	0.5	0.5	0.6	0.4

ICD-10, International Statistical Classification of Diseases and Related Health Problems 10^th^ revision.

## Data Availability

In general, cancer registry data are not publicly available. Anonymised cancer incidence data for Switzerland by cancer site, sex, period, and canton are available at http://www.nicer.org/NicerReportFiles2017/EN/report/atlas.html?&geog=0.

## Supplementary Materials

Supplementary Materials Supplementary Material Table 1: Comparison of number of cases registered for selected types of cancer as published in different annual reports, Cancer Registry Zurich and Zug, Switzerland.

## References

[1] Bray F., Parkin D. M. (2009). Evaluation of data quality in the cancer registry: Principles and methods. Part I: Comparability, validity and timeliness. *European Journal of Cancer*.

[2] Parkin D. M., Bray F. (2009). Evaluation of data quality in the cancer registry: Principles and methods Part II. Completeness. *European Journal of Cancer*.

[3] Bullard J., Coleman M. P., Robinson D., Lutz J.-M., Bell J., Peto J. (2000). Completeness of cancer registration: A new method for routine use. *British Journal of Cancer*.

[4] Dimitrova N., Parkin D. M. (2015). Data quality at the Bulgarian National Cancer Registry: An overview of comparability, completeness, validity and timeliness. *Cancer Epidemiology*.

[5] Larsen I. K., Småstuen M., Johannesen T. B. (2009). Data quality at the Cancer Registry of Norway: An overview of comparability, completeness, validity and timeliness. *European Journal of Cancer*.

[6] Sigurdardottir L. G., Jonasson J. G., Stefansdottir S. (2012). Data quality at the Icelandic Cancer Registry: Comparability, validity, timeliness and completeness. *Acta Oncologica*.

[7] Leinonen M. K., Miettinen J., Heikkinen S., Pitkäniemi J., Malila N. (2017). Quality measures of the population-based Finnish Cancer Registry indicate sound data quality for solid malignant tumours. *European Journal of Cancer*.

[8] Donnelly C., Cairnduff V., Chen J. J. (2017). The completeness and timeliness of cancer registration and the implications for measuring cancer burden. *Cancer Epidemiology*.

[9] Kearney T. M., Donnelly C., Kelly J. M., O'Callaghan E. P., Fox C. R., Gavin A. T. (2015). Validation of the completeness and accuracy of the Northern Ireland Cancer Registry. *Cancer Epidemiology*.

[10] Hackl M., Waldhoer T. (2013). Estimation of completeness of case ascertainment of Austrian cancer incidence data using the flow method. *European Journal of Public Health*.

[11] Fung J. W. M., Lim S. B. L., Zheng H. (2016). Data quality at the Singapore Cancer Registry: An overview of comparability, completeness, validity and timeliness. *Cancer Epidemiology*.

[12] Cendales R., Pardo C., Uribe C., López G., Yepez M. C., Bravo L. E. (2012). Data quality at population-based cancer registries in Colombia. *Biomédica*.

[13] Shimakawa Y., Bah E., Wild C. P., Hall A. J. (2013). Evaluation of data quality at the Gambia national cancer registry. *International Journal of Cancer*.

[14] Dehler S., Tonev S., Korol D., Rohrmann S., Dimitrova N. (2014). Recent trends in cancer incidence: Impact of risk factors, diagnostic activities and data quality of registration. *TUMORI*.

[15] Trama A., Marcos-Gragera R., Sánchez Pérez M. J. (2018). Data Quality in Rare Cancers Registration: The Report of the RARECARE Data Quality Study. *Tumori Journal*.

[16] Lorez M., Bordoni A., Bouchardy C. (2017). Evaluation of completeness of case ascertainment in Swiss cancer registration. *European Journal of Cancer Prevention*.

[17] Parkin D. M., Whelan S. L., Ferlay J., Teppo L., Thomas D. B. (2002). Cancer Incidence in Five Continents Vol. VIII. *IARC Scientific Publications No 155*.

[18] Waterhouse J., Muir C., Correa P., Powell J. (1976). Cancer incidence in five continents, Volume III 1976. *IARC (International Agency for Research on Cancer) Scientific Publications*.

[19] Jensen O. M. (1991). *Cancer Registration, Principles and Methods*.

[20] Tyczynski J. E., DΘmaret E., Parkin D. M. Standards and guidelines for cancer registration in.

[21] Pritchard-Jones K., Kaatsch P., Steliarova-Foucher E., Stiller C., Coebergh J. (2006). Cancer in children and adolescents in Europe: Developments over 20 years and future challenges. *European Journal of Cancer*.

[22] Oberli L. S., Valeri F., Korol D., Rohrmann S., Dehler S. (2016). 31 years of lung cancer in the canton of Zurich, Switzerland: incidence trends by sex, histology and laterality. *Swiss Medical Weekly*.

[23] Pellacani G., Lo Scocco G., Vinceti M. (2008). Melanoma epidemic across the millennium: time trends of cutaneous melanoma in Emilia-Romagna (Italy) from 1997 to 2004. *Journal of the European Academy of Dermatology and Venereology*.

[24] Stang A., Pukkala E., Sankila R., Söderman B., Hakulinen T. (2006). Time trend analysis of the skin melanoma incidence of Finland from 1953 through 2003 including 16,414 cases. *International Journal of Cancer*.

[25] Stang A., Valiukeviciene S., Aleknaviciene B., Kurtinaitis J. (2006). Time trends of incidence, mortality, and relative survival of invasive skin melanoma in Lithuania. *European Journal of Cancer*.

[26] Ferlay J., Steliarova-Foucher E., Lortet-Tieulent J. (2013). Cancer incidence and mortality patterns in Europe: estimates for 40 countries in 2012. *European Journal of Cancer*.

[27] Minini R., Rohrmann S., Braun R., Korol D., Dehler S. (2017). Incidence trends and clinical-pathological characteristics of invasive cutaneous melanoma from 1980 to 2010 in the Canton of Zurich, Switzerland. *Melanoma Research*.

