# Supplementary material for: Indicators of Data Quality at the Cancer Registry Zurich and Zug in Switzerland
Source: Biomed Res Int. 2018 Jun 13;2018:7656197. doi: 10.1155/2018/7656197 (PMC6020656; doi:10.1155/2018/7656197)
Supplement: Supplementary Materials — Supplementary Material Table 1: Comparison of number of cases registered for selected types of cancer as published in different annual reports, Cancer Registry Zurich and Zug, Switzerland. [file 7656197.f1.docx]

Supplementary Material Table 1. Comparison of number of cases registered for selected types of cancer as published in different annual reports, Cancer Registry Zurich and Zug, Switzerland

| **Localisation** | **ICD10** | **Incidence year** | **Annual report 2014**^1)^ | **Annual report 2015**^2)^ | **Annual report 2016**^3)^ | **Diff. 2014-2015** | **%** | **Diff. 2015-2016** | **%** |
| --- | --- | --- | --- | --- | --- | --- | --- | --- | --- |
| **Men** |  |  |  |  |  |  |  |  |  |
| Prostate | C61 | 2012 | 942 | 985 | 997 | 43 | **4.4** | 12 | 1.2 |
|  | C61 | 2013 |  | 925 | 953 |  |  | 28 | **2.9** |
| Lung | C33-C34 | 2012 | 423 | 433 | 435 | 10 | **2.3** | 2 | 0.5 |
|  | C33-C34 | 2013 |  | 393 | 403 |  |  | 10 | **2.5** |
| Colon/Rectum | C18-C20 | 2012 | 361 | 372 | 377 | 11 | **3.0** | 5 | 1.3 |
|  | C18-C20 | 2013 |  | 381 | 389 |  |  | 8 | **2.1** |
| Melanoma | C43 | 2012 | 329 | 330 | 334 | 1 | **0.3** | 4 | 1.2 |
|  | C43 | 2013 |  | 295 | 297 |  |  | 2 | **0.7** |
| Leukaemia | C91-C95 | 2012 | 78 | 87 | 93 | 9 | **10.3** | 6 | 6.5 |
|  | C91-C95 | 2013 |  | 89 | 95 |  |  | 6 | **6.3** |
| Pancreas | C25 | 2012 | 104 | 106 | 106 | 2 | **1.9** | 0 | 0.0 |
|  | C25 | 2013 |  | 116 | 118 |  |  | 2 | **1.7** |
| Liver | C22 | 2012 | 86 | 89 | 91 | 3 | **3.4** | 2 | 2.2 |
|  | C22 | 2013 |  | 68 | 75 |  |  | 7 | **9.3** |
| Urinary tract | C64-C66, C68 | 2012 | 85 | 88 | 90 | 3 | **3.4** | 2 | 2.2 |
|  | C64-C66, C68 | 2013 |  | 119 | 125 |  |  | 6 | **4.8** |
| Bladder | C67 | 2012 | 166 | 170 | 172 | 4 | **2.4** | 2 | 1.2 |
|  | C67 | 2013 |  | 149 | 151 |  |  | 2 | **1.3** |
| **Women** |  |  |  |  |  |  |  |  |  |
| Breast | C50 | 2012 | 1101 | 1107 | 1114 | 6 | **0.5** | 7 | 0.6 |
|  | C50 | 2013 |  | 1034 | 1054 |  |  | 20 | **1.9** |
| Lung | C33-C34 | 2012 | 281 | 290 | 295 | 9 | **3.1** | 5 | 1.7 |
|  | C33-C34 | 2013 |  | 293 | 306 |  |  | 13 | **4.2** |
| Colon/Rectum | C18-C20 | 2012 | 306 | 313 | 315 | 7 | **2.2** | 2 | 0.6 |
|  | C18-C20 | 2013 |  | 341 | 344 |  |  | 3 | **0.9** |
| **Localisation** | **ICD10** | **Incidence year** | **Annual report 2014**^1)^ | **Annual report 2015**^2)^ | **Annual report 2016**^3)^ | **Diff. 2014-2015** | **%** | **Diff. 2015-2016** | **%** |
| Melanoma | C43 | 2012 | 275 | 277 | 279 | 2 | **0.7** | 2 | 0.7 |
|  | C43 | 2013 |  | 258 | 261 |  |  | 3 | **1.1** |
| Leukaemia | C91-C95 | 2012 | 73 | 81 | 84 | 8 | **9.9** | 3 | 3.6 |
|  | C91-C95 | 2013 |  | 77 | 80 |  |  | 3 | **3.8** |
| Pancreas | C25 | 2012 | 116 | 120 | 121 | 4 | **3.3** | 1 | 0.8 |
|  | C25 | 2013 |  | 125 | 131 |  |  | 6 | **4.6** |
| Liver | C22 | 2012 | 40 | 43 | 44 | 3 | **7.0** | 1 | 2.3 |
|  | C22 | 2013 |  | 45 | 48 |  |  | 3 | **6.3** |
| Urinary tract | C64-C66, C68 | 2012 | 60 | 62 | 64 | 2 | **3.2** | 2 | 3.1 |
|  | C64-C66, C68 | 2013 |  | 71 | 72 |  |  | 1 | **1.4** |
| Bladder | C67 | 2012 | 52 | 55 | 56 | 3 | **5.5** | 1 | 1.8 |
|  | C67 | 2013 |  | 47 | 47 |  |  | 0 | **0.0** |
| **Men and women** |  |  |  |  |  |  |  |  |  |
| All tumours | C00-C97 (except C44) | 2012 | 6883 | 7055 | 7154 | 172 | **2.4** | 99 | 1.4 |
|  |  | 2013 |  | 6856 | 7046 |  |  | 190 | **2.7** |

^1)^ published in spring 2015, reports mainly on incidence year 2012

^2)^ published in spring 2016, reports mainly on incidence year 2013

^3)^ published in spring 2017, reports mainly on incidence year 2014
